# Phases of aesthetic judgment in art perception

**DOI:** 10.3389/fpsyg.2026.1785560

**Published:** 2026-04-22

**Authors:** Yuka Nojo

**Affiliations:** Graduate School of Arts and Sciences, The University of Tokyo, Tokyo, Japan

**Keywords:** aesthetic judgment, decision making, EDEN, eye tracking, gaze dynamics, art perception, *Ukiyo-e*

## Abstract

**Background:**

Aesthetic judgment in visual art has often been treated as a final evaluative outcome, but the temporal process through which such judgment emerges during viewing remains insufficiently understood. Although gaze behaviour has been associated with aesthetic evaluation, it is still unclear when and how perceptual exploration becomes linked to judgment formation.

**Methods:**

We examined gaze dynamics during self-paced viewing of 12 Japanese woodblock landscape prints (Ukiyo-e) in 35 participants. Participants observed each artwork until they felt able to make an aesthetic judgment, and then reported their rating. Gaze data from the first 30 s were analysed in successive 3-s time bins using EMHMM-based gaze-state representations and distributional indices, including Hnorm, MaxP, NeffNorm, TV, and Switch.

**Results:**

In the early phase of viewing, gaze behaviour was exploratory and spatially distributed regardless of whether subsequent evaluations were high or low. Clear group differences emerged locally during the middle phase (6–12 s): viewing episodes leading to higher evaluations showed earlier reduction of uncertainty and earlier convergence of gaze-state distributions, whereas lower-evaluation episodes remained more exploratory for longer. However, these effects should be interpreted as exploratory when multiple-comparison correction is taken into account.

**Conclusion:**

These findings suggest that aesthetic judgment is not completed immediately at stimulus onset, but becomes manifest within a temporally unfolding perceptual process. The study supports a framework in which aesthetic judgment is understood as a form of decision-making emerging through the transition from exploration to convergence in gaze dynamics.

## 1 Introduction

Aesthetic judgment in the appreciation of paintings has been widely investigated as a subjective evaluation that viewers form toward a work. Traditionally, aesthetic judgment has been measured through responses to rating scales or forced-choice tasks and has often been treated as a result rendered at the final stage of viewing (Leder et al., 2004; Cupchik, 1995; Silvia, 2005). However, the temporal dimension—namely, how perceptual information is processed during viewing, and at what point and in what manner judgment emerges within this process—has not yet been sufficiently systematised.

Theoretically, the formation process of aesthetic judgment has been organised into stages by Leder et al. (2004). This model assumes a progression consisting of (1) perceptual analysis, (2) implicit memory integration, (3) explicit classification, (4) cognitive mastering, and (5) evaluation, positioning aesthetic evaluation as the cumulative result of perceptual and cognitive processing. However, how these stages unfold within an actual temporal structure during viewing—and, in particular, whether the presence or absence of an evaluative requirement influences this progression—has not been adequately examined through time-resolved analyses of gaze behaviour.

Since 2015, research examining the relationship between gaze behaviour and aesthetic evaluation in art appreciation has advanced substantially. Empirical studies using eye-tracking have reported associations between initial fixation location or fixation duration and preference judgments (Goller et al., 2019; Mitrovic et al., 2020; Pelowski et al., 2016), suggesting that gaze behaviour may be linked to the formation of evaluation. In terms of research design, many studies have investigated early gaze biases under short presentation conditions limited to 2–5 s (Goller et al., 2019), or have required immediate preference judgments after 3–10 s of observation (Mitrovic et al., 2020). Furthermore, in two-alternative forced-choice tasks employing repeated presentations on the order of several hundred milliseconds, a gaze cascade effect has been identified, whereby the probability of fixation on the chosen stimulus increases immediately prior to decision (Shimojo et al., 2003; Glaholt and Reingold, 2009). Some studies have employed presentation durations of approximately 8–15 s (Locher et al., 2007; Massaro et al., 2012; Leder and Nadal, 2014) to examine the relationship between gaze distribution and evaluation. Nevertheless, overall, presentation durations of less than 20 s predominate, and designs that require evaluative responses during viewing or immediately after stimulus offset remain the mainstream approach.

As a recent theoretical framework, the VIMAP (Vienna Integrated Model of Art Perception) model has been proposed by Pelowski et al. (2017). VIMAP conceptualises aesthetic experience as an interaction between prediction/expectation and perceptual information, emphasising a dynamic process that extends from early (approximately 6–8 s) automatic perceptual processing to cognitive reappraisal and emotional response. Within this model, mismatches between the viewer’s predictions and actual perceptual input are assumed to alter attentional allocation and exploratory behaviour, thereby influencing final evaluation. Thus, aesthetic judgment is understood not as a static outcome but as a temporally unfolding process of adjustment. Although recent theoretical models such as VIMAP emphasise the temporal unfolding of aesthetic experience, it remains unclear at which specific phases such dynamics emerge during actual viewing. This gap motivates the present study.

These studies have provided important findings demonstrating the association between gaze and preference. At the same time, in many studies gaze indices are treated as averages across the entire viewing period (Locher et al., 2007; Goller et al., 2019), and relatively few investigations have taken as their primary focus the temporal unfolding itself—namely, at which point during viewing evaluation-related changes occur. Moreover, systematic comparisons between free-viewing conditions and time-limited conditions, examining how the presence or absence of evaluative demands affects the temporal structure of gaze dynamics, remain limited.

Recently, studies employing approximately 30 s of conditionally free viewing have also been reported (Nojo and Chan, 2025; Nojo, 2026). RAIC (Reading an Artist’s Intention from the Composition) demonstrated the relationship between compositional understanding and the organisation of gaze, while EDEN (Early Depth Engagement in Art Perception) reported branching patterns in access to depth cues during the early phase of viewing. These studies provide a foundation for clarifying the temporal unfolding of gaze during the appreciation process. Although aesthetic judgment and preference judgment are not completely identical psychological processes, it has been shown that both share gaze dynamics related to value-based judgment; accordingly, these findings are referenced in the present study in a supplementary manner.

However, these prior investigations have focused on compositional understanding or depth perception, and have not directly examined the temporal structure under which aesthetic judgment itself is established. In particular, it remains insufficiently clarified how the temporal structure of gaze dynamics differs between conditions in which an explicit instruction such as “please judge the beauty” is given, and conditions in which evaluative demands are separated from stimulus presentation.

In light of this perspective, the present study investigates how gaze organisation evolves across time during extended viewing. Under conditions in which judgment is externally enforced, the evaluation stage in the Leder model may become foregrounded at an early phase, and the linkage between gaze and evaluation may emerge relatively early. By contrast, under conditions in which the timing of judgment is entrusted to the viewer, stages such as perceptual analysis and cognitive mastering may proceed independently of evaluation, and the temporal organisation of gaze may precede explicit judgment. In other words, the presence or absence of task demands itself may alter the temporal structure of this staged progression.

In the present study, we employ a self-paced free-viewing condition that does not require an immediate response on a rating scale, but imposes only the minimal constraint that participants respond when their evaluation of beauty has been determined. Under conditions in which the timing of judgment is not externally specified during stimulus presentation, we analyse the temporal transitions of gaze dynamics before and after the establishment of judgment in order to examine through what perceptual processes aesthetic judgment emerges. Furthermore, we introduce, in a supplementary manner, a condition in which the timing of judgment is externally constrained, and compare how the presence or absence of task demands influences the temporal structure of gaze. Although aesthetic judgment and preference judgment are not fully identical psychological processes, both have been shown to share gaze dynamics related to value-based evaluation, and thus are referred to here in a supplementary capacity.

Building upon recent findings in gaze × preference research and the theoretical framework of the Leder model, the present paper focuses on differences in task structure—specifically, the presence or absence of evaluative demands—and aims to explicitly position the temporal formation process of aesthetic judgment from the perspective of gaze dynamics.

## 2 Previous studies

Research on aesthetic judgment has largely centred on identifying the cognitive and neural correlates associated with reported evaluation values (Jacobsen et al., 2004; Leder et al., 2004; Pelowski et al., 2016; Chatterjee and Vartanian, 2014). While rating-based and choice-based paradigms have yielded valuable insights into evaluative outcomes, comparatively less attention has been devoted to modelling the internal temporal dynamics through which perceptual processing progressively develops into a stable judgment state. As a result, empirical investigations have tended to emphasise reported evaluation values, while the dynamic progression through which perceptual engagement develops into judgment has received comparatively less direct examination.

By contrast, in recent cognitive science and decision-making research, judgment has been conceptualised not as an instantaneous choice outcome, but as a process formed through the temporal accumulation of perceptual information and changes in internal states (Bogacz et al., 2006; Gold and Shadlen, 2007; Ratcliff and McKoon, 2008; Krajbich et al., 2010). For example, sequential evidence accumulation models demonstrate that decisions are formed as temporal processes that unfold until a given threshold is reached. This framework underscores the importance of examining the temporal structure of judgment, including in the domain of aesthetic evaluation (Ratcliff and McKoon, 2008; Gold and Shadlen, 2007).

It has been repeatedly reported that gaze behaviour during the viewing of paintings is closely associated with aesthetic evaluation and preference judgment (Locher et al., 2001; Locher, 2012; Goller et al., 2019; Mitrovic et al., 2020). Indices such as fixation distribution, dwell time, and revisitation behaviour during viewing have been suggested to reflect the degree of engagement with a work and tendencies in evaluation (Locher et al., 2007; Smith et al., 2017). These findings indicate that gaze behaviour is not merely a by-product of perceptual input, but constitutes a dynamic process that is reciprocally related to judgment formation.

However, many eye-tracking studies have primarily relied on aggregated metrics computed over the entire viewing period (Locher, 2012; Goller et al., 2019; Mitrovic et al., 2020). Consequently, the fine-grained temporal transitions in gaze organisation that precede the consolidation of evaluation remain under-characterised at an empirical level.

Research using choice paradigms has demonstrated that preference or value judgments toward visual stimuli can manifest as gaze biases from very early after stimulus onset. In particular, in two-alternative forced-choice tasks, gaze biases consistent with the subsequent choice have been observed immediately following stimulus presentation, a phenomenon known as the gaze cascade effect (Shimojo et al., 2003; Glaholt and Reingold, 2009). These findings support the view that judgments can be directionally biased at very early time scales.

Nevertheless, such studies are based primarily on conditions in which the timing of judgment is externally constrained or on task settings in which comparisons between stimuli are explicitly required, and therefore differ from situations in which viewers establish judgments at their own timing. Moreover, compared with simple images or consumer products that are frequently used as stimuli, it remains insufficiently examined how similar temporal structures manifest in artworks, which typically contain a high density of information and require semantic interpretation (Leder and Nadal, 2014).

In light of these prior findings, at least two aspects must be distinguished when examining the temporal structure of aesthetic judgment. One concerns the extent to which judgments can be directionally biased under conditions in which evaluation is externally required; the other concerns the processes through which judgments are formed under conditions in which the timing of judgment is entrusted to the viewer.

With respect to the latter in particular, existing research has rarely implemented event-aligned analyses anchored to the moment of decision, thereby limiting insight into how perceptual integration evolves immediately prior to judgment consolidation. Accordingly, under self-paced viewing conditions, it remains unclear how gaze dynamics relate to subsequent evaluation.

The present paper is characterised by its use of a free-viewing condition in which the timing of judgment is entrusted to the participant, and by its examination of the temporal process leading to the establishment of aesthetic judgment through transitions in gaze behaviour. Whereas existing research has focused primarily on judgment outcomes or early directional biases, the present investigation aims to achieve a more comprehensive understanding of the temporal structure of aesthetic judgment by targeting the process leading up to judgment establishment itself.

## 3 Materials and methods

### 3.1 Participants

Thirty-five individuals participated in the experiment. For all participants, both the time of establishment of aesthetic judgment and gaze behaviour data during viewing were successfully recorded, and all 35 participants were included in the analysis (22 males, 13 females; mean age = 21.7 years, SD = 4.4). A within-subjects design was employed, and multiple trials of judgment data as well as gaze time-series data were collected from each participant. In addition to the main experiment, an auxiliary experiment was conducted using a separate group of participants who performed a two-alternative forced-choice preference task, in order to examine gaze behaviour immediately prior to decision-making. All participants had normal or corrected-to-normal vision and provided informed consent prior to participation in the experiment. None of the participants had received formal professional training in art. The study was conducted with the approval of the ethics committee of the university to which the first author is affiliated. Upon completion of the experiment, participants received monetary compensation in accordance with institutional guidelines.

### 3.2 Stimuli

In the present experiment, twelve *Ukiyo-e* works that had been employed in prior research were used as stimuli. These works were selected on the basis of a preliminary experiment that examined a total of 101 *Ukiyo-e* artworks; the detailed selection procedure and the distribution of evaluation values have been reported previously (Nojo et al., 2023). Each work consisted of a landscape painting by Katsushika Hokusai or Utagawa Hiroshige, and the same set of stimuli has also been used in research on RAIC (*Reading an Artist’s Intention from the Composition*; Nojo and Chan, 2025) as well as in research on depth perception (Nojo, 2026). Within the present paper, the artworks are referred to by abbreviated titles (e.g., *GreatWave*, *Ejiri*) (see Supplementary Figure 1 and Supplementary Table 1 for details). Accordingly, the twelve works used in the present experiment were selected in advance from a broad stimulus pool and do not constitute an arbitrary selection of stimuli. In the study on depth perception (Nojo, 2026), a phenomenon termed EDEN (*Early Depth Engagement in Art Perception*) was reported for these works, in which access to depth-related regions temporally diverges during the early phase of viewing.

### 3.3 Procedure

Eye movements were recorded using a Tobii Pro TX300 eye tracker (Tobii AB, Stockholm, Sweden). Stimuli were presented on a 23-inch monitor with a resolution of 1920 × 1,080 pixels, and gaze data were sampled at 300 Hz. Stimulus presentation and data acquisition were controlled using Tobii Pro Lab. Participants’ viewing distance was fixed at approximately 52 cm by means of a chin rest. Prior to the start of the experiment, a five-point calibration procedure was conducted, and binocular data were recorded. Preprocessing and analyses were performed using custom scripts written in MATLAB. For each stimulus, participants were asked to provide an aesthetic judgment on a 10-point scale ranging from 1 to 10. Each trial began with the presentation of a central fixation point (a cross) on a white background for 5 s. Subsequently, the stimulus image was presented without a fixed time limit. Participants were instructed to click the mouse at the moment they judged that they were able to make a 10-point aesthetic evaluation. Upon clicking, the display transitioned to the response screen, where participants selected the corresponding evaluation value using a mouse-operated selection button. This condition differs from a fixed 30-s free-viewing condition in that the timing of evaluation establishment was entrusted to the participant, constituting a self-paced viewing condition. This design enables the direct examination of the temporal progression leading up to the establishment of judgment.

## 4 Data analysis

### 4.1 ROI extraction using EMHMM

The preprocessing of gaze data and the procedure for extracting Regions of Interest (ROIs) in the present analysis were fundamentally identical to those employed in prior research. ROI extraction was conducted using the Eye Movement Hidden Markov Model (EMHMM; Chuk et al., 2014; Hsiao et al., 2021). EMHMM is a probabilistic model that simultaneously estimates the spatial fixation distribution and its temporal transition structure from gaze sequences of multiple observers. A key characteristic of this method is that it enables the data-driven extraction of representative gaze states (ROIs).

For each stimulus image, participants’ gaze sequences (x, y coordinates) were used as input, and fixation states (ROIs), represented as Gaussian distributions, as well as the transition probabilities among these states, were estimated simultaneously. To avoid effects arising from differences in image size, all stimulus images and gaze coordinates were normalised to a common resolution.

The number of ROIs was fixed at 14 states. This decision was based on preliminary analyses showing that improvements in model likelihood associated with increasing the number of states asymptotically saturated, such that further subdivision of states did not yield interpretative gains. In addition, a comparable number of states has been employed in prior studies as a stable representation of gaze behaviour. During model learning, multiple initialisations were performed to reduce the influence of dependence on initial parameter values, and the solution yielding the maximum log-likelihood was adopted. Furthermore, to prevent the emergence of excessively large or excessively small ROIs, statistical constraints were imposed on ROI area and axis length. Specifically, ROI area was restricted to a range of 0.1–5% of the total image area, and ROI axis lengths were clamped based on the mean ± Nσ to ensure that they did not deviate markedly from the overall ROI distribution. Each ROI was represented as a two-dimensional Gaussian distribution characterised by a mean location and variance.

### 4.2 Inference of gaze state sequences

Using the ROIs (*K* = 14) estimated by EMHMM in the preceding stage as the state space, the continuous gaze time-series data were converted into discrete gaze-state sequences for each participant and each artwork. The observation sequence was defined as the gaze coordinates at each time point, and the states were defined as the ROIs specified by EMHMM. Using the trained HMM parameters, we estimated, for each time point, the posterior probability of belonging to each state as well as the most likely state sequence (the Viterbi path). Through this procedure, each gaze trajectory was represented as a temporal progression of gaze states.

### 4.3 Aggregation of state distributions within time bins

The estimated gaze-state sequences were aggregated by dividing the first 30 s from viewing onset into time bins of 3-s width. Within each time bin, we computed (1) the mean dwell-probability distribution across states, (2) the number of visits to each state (hit count), and (3) the total dwell time within each state (dwell time). Through these procedures, each time bin was quantitatively characterised as a gaze-state distribution corresponding to that temporal segment.

### 4.4 Measures of uncertainty and concentration of gaze distribution

In the present study, gaze behaviour is conceptualised not merely in terms of fixation counts or dwell time, but as the temporal organisation of the gaze-state distribution. Accordingly, the structure of the state distribution within each time bin was quantified from the perspectives of exploratoriness and convergence. Here, distributional uncertainty (*Hnorm*) indicates the extent to which gaze is dispersed across multiple states: higher values correspond to an exploratory allocation of gaze, whereas lower values indicate convergence toward a specific state. By contrast, *MaxP* captures the degree of concentration on the most dominant state and serves as an index for evaluating gaze dominance. *NeffNorm* reflects the effective number of states that are substantively utilised, thereby indexing the breadth of exploration. By jointly using these indices, it becomes possible to capture, in a time-resolved manner, the process by which gaze transitions from an exploratory phase to a phase of organisation and convergence.

To evaluate the uncertainty and concentration of the gaze-state distribution within each time bin, the present study aims to characterise gaze dynamics associated with the formation of aesthetic judgment from two complementary aspects: (1) distributional structure (*Hnorm, MaxP, NeffNorm*) and (2) temporal reorganisation (*TV, Switch*).

State entropy was calculated as


$$\text{Hnorm}=-\sum_{i=1}^{K}p_{i}\log p_{i}$$

and a normalised index (*Hnorm*) was obtained by dividing by the theoretical maximum value *log K*. Here, *p_i_* denotes the mean posterior probability of state *i* within a given time bin. Higher values of *Hnorm* indicate that the gaze-state distribution is more dispersed, reflecting an exploratory state in which multiple ROIs are utilised relatively evenly. Conversely, lower values of *Hnorm* indicate that probability mass is concentrated on specific states, reflecting a convergent and organised gaze state.

From the same state distribution, the maximum state probability (*MaxP*) was calculated in order to evaluate the strength of the most dominant gaze state within the given time bin. Higher values of *MaxP* indicate that a single ROI is predominantly utilised, reflecting gaze concentration and stabilisation. In contrast, lower values of *MaxP* indicate smaller probability differences among states and a more dispersed allocation of gaze.


$$\text{MaxP}=\max_{i}p_{i}$$

Furthermore, the entropy-based effective number of states [*Neff* = exp.(*H*)] and its normalised form with respect to the number of ROIs (NeffNorm) were computed. Lower values indicate that only a small number of ROIs are effectively utilised, reflecting convergence of gaze toward specific states.


$$N_{\mathrm{eff}}\text{Norm}=\exp(H)/K$$

Total Variation (*TV*) was calculated as the difference between gaze-state distributions in consecutive time bins t and t + 1, defined as follows:


$$\mathrm{TV}(t)=\frac{1}{2}\sum_{i=1}^{K}|p_{i}(t+1)-p_{i}(t)|$$

Here, *pi(t)* denotes the mean posterior probability of state *i* in time bin *t*. *TV* ranges from 0 to 1, with higher values indicating greater changes in the gaze-state distribution between consecutive time bins. Thus, high*TV* reflects a temporally unstable and actively reorganising state, whereas low *TV* reflects a stable distribution with minimal change.

In addition, based on the most likely state sequence (Viterbi path), the state-switching frequency per unit time (Switch rate) was computed. *Switch* is an index representing how frequently gaze transitions occur between different ROIs. Higher *Switch* values indicate frequent transitions between states, reflecting an exploratory and unstable state. In contrast, lower *Switch* values indicate longer dwell within the same state and stable maintenance of gaze.


$$\text{Switch}=\frac{1}{T-1}\sum_{t-1}^{T-1}1T(S_{t}\neq S_{t}+1)$$

### 4.5 Dynamic properties of gaze transitions

To capture temporal changes in the gaze-state distribution, the difference between state distributions across consecutive time bins was computed as Total Variation (*TV*). *TV* quantifies the magnitude of change in the state distribution between adjacent time bins and indexes the degree of reorganisation in gaze organisation. Higher values indicate that the distributional structure is changing substantially, whereas lower values indicate that the distribution has stabilised. In addition, the state-switching frequency (Switch rate) in the most likely state sequence (Viterbi path) was computed to evaluate the extent of exploratory gaze switching.

### 4.6 Statistical comparison between aesthetic judgment groups

To define artwork-level aesthetic evaluation groups, we first computed, for each artwork, the mean aesthetic rating after within-participant normalisation of evaluation scores. Based on these mean values, the six artworks with the highest scores were classified as the high-evaluation group, and the six artworks with the lowest scores were classified as the low-evaluation group. This grouping fully corresponded to the classification obtained independently in a conditionally free-viewing experiment under a fixed 30-s presentation condition (Nojo et al., 2023), and was therefore adopted as a common group definition in the subsequent analyses.

Each gaze index was calculated for each artwork and each time bin, and was subsequently compared between the high-evaluation and low-evaluation groups defined on the basis of subjective aesthetic ratings. For between-group comparisons, non-parametric tests were employed, and in addition to *p* values, effect sizes (rank-biserial correlation) were reported.

### 4.7 Decision-proximal gaze analysis in preference tasks

In the preference judgment task, gaze data were temporally aligned to the decision time point, and the probability of gaze directed toward the option that was ultimately chosen was computed as a function of normalised time to decision. To focus on gaze dynamics immediately prior to judgment, the analysis was restricted to a fixed time bin immediately preceding the response (see Supplementary Methods for details).

## 5 Result

In the present study, we compared a high-evaluation group (6 artworks) and a low-evaluation group (6 artworks), which had been predefined on the basis of aesthetic ratings. The 30-s viewing period was divided into 3-s time bins, and the temporal trajectories of gaze-state distribution indices were examined. The primary focus was to determine at which temporal phase aesthetic evaluation would manifest as a divergence in gaze dynamics.

The mean time of establishment of beauty judgment across artworks was 13.1 s (*SD* = 1.21, range = 10.55–14.83 s) (see Supplementary Figure 1 for artwork-specific values).

A group difference was confirmed at the artwork level in aesthetic evaluation values (Wilcoxon rank-sum test, *p* = 0.0022, *z* = 2.88, rank-biserial *r* = 0.83). The mean normalised evaluation value was 0.668 for the high-evaluation group and 0.357 for the low-evaluation group.

### 5.1 Early phase (0–6 s)

During the early viewing phase (0–3 s and 3–6 s), no clear differences were observed between the high- and low-evaluation groups in state-distribution uncertainty (*Hnorm*), effective number of states (*NeffNorm*), or maximum state probability (*MaxP*). Group differences were small for all indices, and no significant divergence was observed in dynamic indices (*TV, Switch*) (all *p* > 0.10). Thus, immediately after viewing onset, no systematic gaze divergence corresponding to aesthetic evaluation was detected.

### 5.2 Middle phase (6–12 s): emergence of evaluation differences

Between 6 and 9 s, group differences were first clearly observed. State-distribution uncertainty (Hnorm) was higher in the low-evaluation group (*p* = 0.038, *r* = −0.185), and the magnitude of distributional change (TV) was also higher in the low-evaluation group (*p* = 0.0067, *r* = −0.242). A similar pattern persisted between 9 and 12 s, with higher values in the low-evaluation group for Hnorm (*p* = 0.0189, *r* = −0.207) and TV (*p* = 0.0076, *r* = −0.235).

In contrast, no clear group differences were observed for *NeffNorm, MaxP,* or *Switch*.

**Figure 1:**
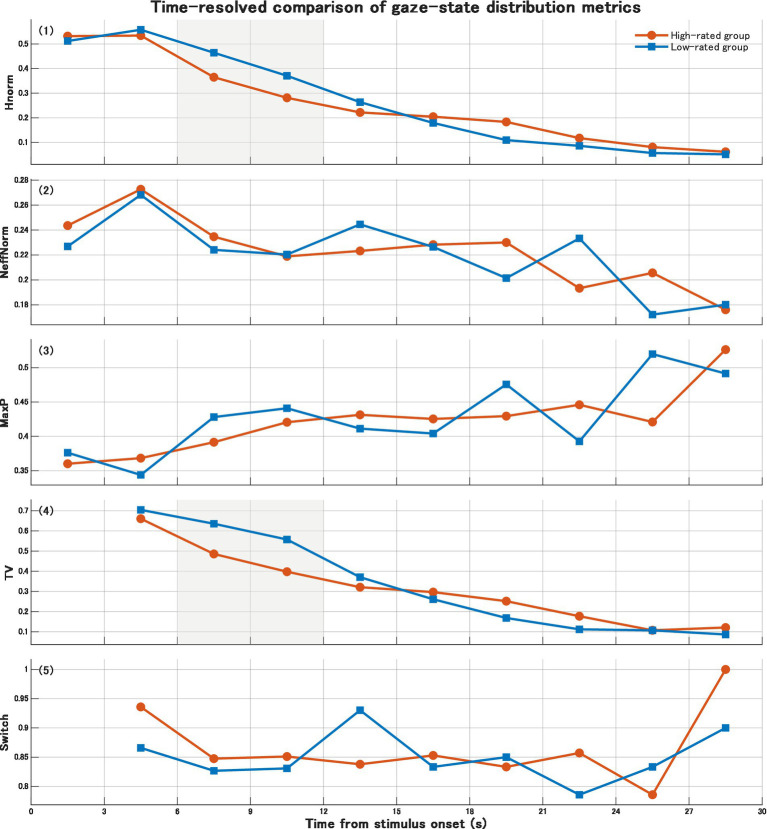
Time-resolved comparison of gaze-state distribution metrics between high- and low-rated artwork groups. Mean values of Hnorm, NeffNorm, MaxP, TV, and Switch are shown across successive 3-s time bins from stimulus onset. Shaded regions indicate the middle viewing phase, during which local group differences were observed before correction.

These results suggest that during the middle viewing phase (6–12 s), viewing that ultimately leads to higher evaluation may be characterised by an earlier reduction in distributional uncertainty and a more rapid progression toward convergence (Figure 1).

### 5.3 Late phase (12–30 s)

After 12 s (12–30 s), no consistent group differences were detected in *Hnorm, NeffNorm, MaxP, TV*, or *Switch* (all *p* > 0.05). Although trends were observed in some individual time bins, no stable divergence was confirmed. Accordingly, evaluation-related differences in the present study were primarily localised to the middle phase (6–12 s; Figure 1).

### 5.4 Correction for multiple comparisons

**Figure 2:**
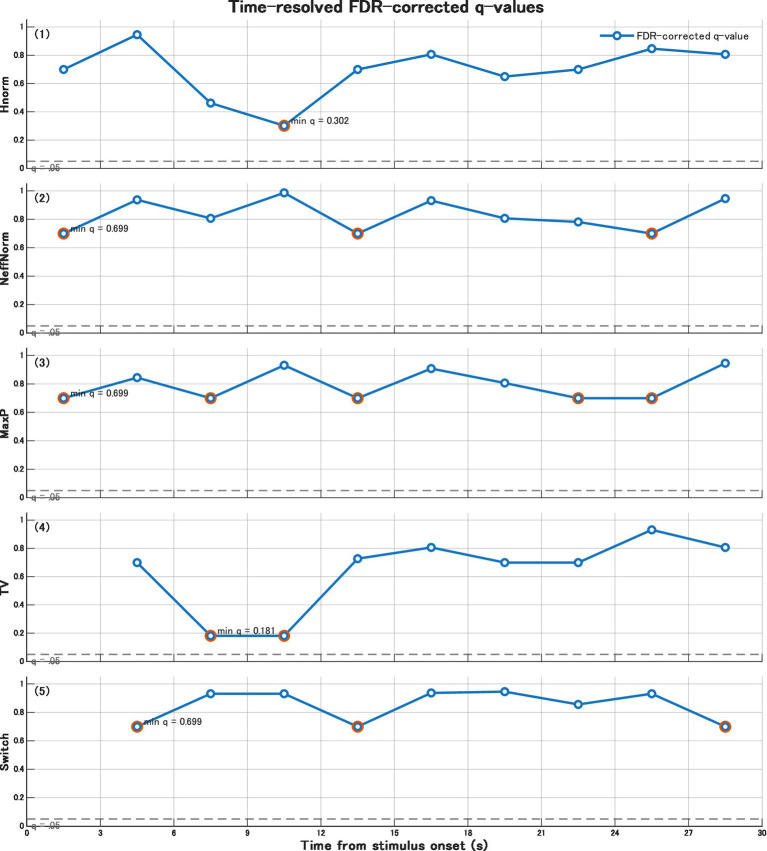
Time-resolved FDR-corrected q-values across time bins and gaze-state metrics. Benjamini–Hochberg false discovery rate correction was applied to all time bin × metric comparisons. No comparison met the criterion of q < 0.05. The smallest q-values were observed for TV in the 6–9 and 9–12 s windows, followed by Hnorm.

False discovery rate (FDR; Benjamini–Hochberg) correction was applied to comparisons across each time bin × index. No differences met the criterion of *q* < 0.05. The smallest q value was observed for TV in the 6–9 and 9–12 s windows (*q* = 0.181), followed by Hnorm in the same time ranges. Therefore, although group differences observed between 6 and 12 s were significant without correction, they cannot be regarded as confirmatory when multiple comparisons are taken into account and should be interpreted as exploratory findings (Figure 2).

### 5.5 Depth-related fixation indices (supplementary analysis)

Independently of state-distribution indices, fixation behaviour directed toward depth-related points (HitCount, FirstHitTime, MeanDwell) was also compared across time bins. At the level of single bins, no stable group differences were observed. An exception was detected for HitCount in the 0–3 s window; however, the effect size was practically small and did not constitute a consistent feature.

By contrast, when bins were cumulatively aggregated, Cumulative HitCount consistently showed group differences from 0–6 s onward (*p* ≈ 0.001–0.007). This pattern did not reflect an instantaneous difference, but rather a tendency that became manifest only in the accumulated total amount of fixation over time. However, no significant differences were confirmed at the participant level, suggesting that this effect reflects a tendency at the group level.

### 5.6 Pre-decision gaze bias (supplementary analysis)

In the preference judgment task, a gaze bias toward the ultimately chosen option was observed immediately prior to decision. Even under strict presentation constraints of 0.3 s or 0.9 s, an increase in gaze directed toward the selected option was confirmed within the time bin immediately preceding the response (Supplementary Figures 2–4).

### 5.7 Summary of results

The principal finding of the present study is that gaze divergence between evaluation groups did not emerge during the early viewing phase, but appeared locally during the middle phase (6–12 s). In particular, in indices of state-distribution uncertainty and distributional change, the high-evaluation group exhibited an earlier tendency toward convergence. In contrast, no stable group differences were observed in the late phase. When multiple comparison correction was taken into account, these effects should be interpreted as exploratory findings.

Taken together, the results suggest that gaze dynamics associated with aesthetic judgment may not become manifest immediately after viewing onset, but rather during the middle phase of viewing.

## 6 General discussion

The present study is characterised by its examination of when and how aesthetic judgment is formed during the viewing process, specifically through gaze dynamics under the explicit task requirement to “judge beauty.” In prior research, presentation durations were typically limited to several seconds to approximately a dozen seconds, and evaluative responses were requested either during viewing or immediately after stimulus offset. Consequently, the relationship between gaze and evaluation has most often been reported in terms of averaged indices, and the temporal unfolding itself—namely, at which phase of viewing evaluation-related changes emerge—has not necessarily been made explicit. By employing time-resolved indices of gaze-state distributions, the present study examined changes in gaze structure associated with the formation of aesthetic judgment. The central concern of this paper lies in the temporal structure of aesthetic judgment; depth cues (EDEN) are referenced only as a supplementary explanatory element.

The results indicate that under conditions in which an evaluative requirement is present, gaze dynamics diverge between evaluation groups during the middle phase of viewing (approximately 6–12 s). Specifically, in viewing episodes that culminated in higher evaluations, uncertainty in the gaze-state distribution (*Hnorm*) decreased earlier, and the magnitude of distributional change (*TV*) showed a relatively rapid convergence. By contrast, in viewing episodes that culminated in lower evaluations, the exploratory state was maintained for a relatively longer duration, and convergence toward specific states was delayed. These findings suggest that aesthetic judgment may proceed not as a single instantaneous decision, but as a process involving a transition from exploration to convergence.

Importantly, stabilisation of gaze per se can occur even under conditions in which no evaluative requirement is imposed. Gaze stabilisation under free-viewing conditions has been widely reported as a general process of perceptual integration (e.g., Locher et al., 2007) and is not specific to the stimuli used in the present study. In a separate 30-s free-viewing condition, a temporal structure was observed in which the gaze-state distribution stabilised at approximately 12 s; however, the degree of stabilisation was not systematically associated with evaluation. In contrast, under the explicit aesthetic judgment condition employed in the present study, the stabilisation process of gaze diverged as a function of evaluation. Thus, while gaze stabilisation may arise as a natural perceptual organisation process, whether such stabilisation becomes differentiated by evaluation may depend on task demands.

This finding suggests that the relationship between gaze and evaluation is not fixed, but may be constructed by the structure of the task. When evaluation is explicitly required, viewers may adjust their exploratory allocation of gaze toward convergence at an earlier stage, thereby advancing information integration consistent with value judgment. Conversely, when the evaluative requirement is separated from stimulus presentation, gaze may stabilise in accordance with perceptual integration processes, but the strength of convergence does not necessarily reflect differences in evaluation. In this manner, the temporal structure linking aesthetic judgment and gaze dynamics may vary depending on whether judgment is foregrounded as an explicit task.

Consideration must also be given to the characteristics of the stimuli. In the *Ukiyo-e* landscape paintings used in the present study, group differences were observed in access to depth-related regions during the early phase of viewing (0–3 s). Prior research on EDEN (Nojo, 2026) reported that modes of access to depth cues can diverge within several seconds after viewing onset. The early differences observed in the present study are unlikely to directly indicate the establishment of evaluation itself; rather, they more plausibly reflect individual differences in attentional selection regarding which information is preferentially incorporated. Access to depth cues does not constitute a sufficient condition for aesthetic judgment, but may be positioned as one of the initial conditions that can influence subsequent information integration processes.

From a theoretical perspective, the stage model proposed by Leder et al. (2004) organises aesthetic processing as proceeding from perceptual analysis to implicit memory integration and explicit classification, ultimately culminating in evaluation. The present findings suggest that these stages may not necessarily unfold in a fixed temporal order. Under free-viewing conditions, the transition from exploration to convergence progressed independently of evaluation, and no clear temporal divergence corresponding to evaluation was observed. By contrast, under conditions in which evaluation was explicitly required, gaze dynamics diverged between evaluation groups during the 6–12 s interval, suggesting that the evaluation stage may be involved earlier in the integration process. In other words, evaluation is not invariably positioned as a terminal stage; rather, depending on task context, it may be foregrounded and exert influence on upstream perceptual integration processes. This implies that the temporal arrangement of stages in the model may be reorganised by task structure, indicating the potential plasticity of the stage architecture. However, the temporal divergence observed in the present study should be interpreted as exploratory. More refined examination of the temporal coupling between gaze dynamics and aesthetic judgment will require direct within-participant manipulation of the presence or absence of evaluative demands, as well as analyses temporally aligned to the moment of judgment establishment. Future research that systematically compares the interaction between task structure and gaze organisation may further clarify the temporal formation mechanisms of aesthetic judgment.

In summary, the present study provides empirical evidence suggesting that aesthetic judgment does not arise instantaneously at a fixed point in time, but is formed within a temporal process characterised by a transition from exploration to convergence, and that this process may be dynamically shaped by task demands. The findings indicate that aesthetic judgment is temporally constructed through the interaction between perceptual processes and task context, and that the presence or absence of an evaluative requirement may fundamentally alter the interpretation of gaze dynamics. In this respect, the present study offers a new perspective to the field of aesthetic evaluation research.

## 7 Limitations and future direction

The findings of the present paper were obtained under the specific characteristics of the *Ukiyo-e* stimuli employed in this study, and further investigation is required to determine the extent to which these results generalise to other artistic styles or compositional conditions. Because physical properties such as composition, colour, and visual saliency were not systematically controlled across stimuli, it cannot be ruled out that certain aspects of the observed gaze dynamics may depend on stimulus-specific characteristics.

In addition, the present study did not conduct analyses time-locked to the moment of judgment. To capture more directly the gaze dynamics immediately preceding the establishment of judgment, future research should implement event-locked temporal alignment analyses as an important next step.

## 8 Conclusion

The present study demonstrated that aesthetic judgment does not become immediately fixed at the very onset of viewing, but may instead become manifest within the temporal progression of gaze dynamics. During the early viewing phase (0–3 s), gaze behaviour remained exploratory even under conditions in which evaluation was explicitly required as a task, and no evidence was obtained to indicate that aesthetic evaluation had stably emerged at this stage. On the other hand, the observation of group differences in fixation toward depth-related regions during the early phase suggests that information selection relevant to judgment formation may diverge from an early point in time. However, these early differences do not directly demonstrate the establishment of evaluation itself.

During the middle viewing phase (6–12 s), uncertainty in the gaze-state distribution and the magnitude of distributional change diverged between evaluation groups, and viewing episodes leading to higher evaluations exhibited an earlier tendency toward convergence. Importantly, although gaze stabilisation per se can occur even under conditions without evaluative demands, the present findings suggest that when aesthetic judgment is explicitly required, as in this study, the stabilisation process may be reconfigured in a manner that incorporates evaluation differences. In other words, the temporal organisation of gaze may proceed as a natural perceptual integration process, yet whether such organisation becomes linked to value differentiation may depend on task structure.

Although the present results partially correspond to the temporal scale reported in research on depth perception, what was directly examined here was not the establishment of perception itself, but the process by which perceived information acquires evaluative meaning. Early access to depth cues does not constitute a sufficient condition for aesthetic judgment; however, it may contribute to a dynamic causal process during subsequent information integration processes.

Taken together, the present paper supports a perspective that conceptualises aesthetic judgment not as a simple immediate response, but as a process that becomes manifest within the temporal structure of gaze dynamics transitioning from exploration to convergence, through interaction with task demands. The relationship between evaluation and gaze is not a fixed correspondence; rather, it may become clearly linked only within contexts in which judgment is foregrounded. The present study provides an empirical foundation for understanding the temporal formation of aesthetic judgment within the interaction between perceptual processes and task structure.

## Data Availability

The datasets presented in this study are publicly available in OSF at: https://osf.io/bek3h/overview?view_only=29a7aa312d874616908cfe00d8c30231.

## References

[ref1] Bogacz R. Brown E. Moehlis J. Holmes P. Cohen J. D. (2006). The physics of optimal decision making: a formal analysis of models of performance in two-alternative forced-choice tasks. Psychol. Rev. 113, 700–765. doi: 10.1037/0033-295X.113.4.700, 17014301

[ref2] Chatterjee A. Vartanian O. (2014). Neuroaesthetics. Trends Cogn. Sci. 18, 370–375. doi: 10.1016/j.tics.2014.03.003, 24768244

[ref9001] Chuk T. Chan A. B. Hsiao J. H. (2014). Understanding eye movements in face recognition using hidden Markov models. J. Vis. 14:8. doi: 10.1167/14.11.8 25228627

[ref3] Cupchik G. C. (1995). Emotion in aesthetics: reactive and reflective models. Poetics 23, 177–188. doi: 10.1016/0304-422X(94)00014-W

[ref4] Glaholt M. G. Reingold E. M. (2009). The time course of gaze bias in visual decision tasks. Vis. Cogn. 17, 1228–1243. doi: 10.1080/13506280802362962

[ref5] Gold J. I. Shadlen M. N. (2007). The neural basis of decision making. Annu. Rev. Neurosci. 30, 535–574. doi: 10.1146/annurev.neuro.29.051605.113038, 17600525

[ref6] Goller J. Mitrovic A. Leder H. (2019). Effects of liking on visual attention in faces and paintings. Acta Psychol. 197, 115–123. doi: 10.1016/j.actpsy.2019.05.008, 31146088

[ref9002] Hsiao J. H. Lan H. Zheng Y. Chan A. B. (2021). Eye movement analysis with hidden Markov models (EMHMM) with co-clustering. Behav. Res. Methods 53, 2473–2486. doi: 10.3758/s13428-021-01541-5 33929699 PMC8613150

[ref7] Jacobsen T. Buchta K. Köhler M. Schröger E. (2004). The primacy of beauty in judging the aesthetics of objects. Psychol. Rep. 94, 1253–1260. doi: 10.2466/pr0.94.3c.1253-1260, 15362400

[ref8] Krajbich I. Armel C. Rangel A. (2010). Visual fixations and the computation and comparison of value in simple choice. Nat. Neurosci. 13, 1292–1298. doi: 10.1038/nn.2635, 20835253

[ref9] Leder H. Belke B. Oeberst A. Augustin D. (2004). A model of aesthetic appreciation and aesthetic judgments. Br. J. Psychol. 95, 489–508. doi: 10.1348/0007126042369811, 15527534

[ref10] Leder H. Nadal M. (2014). Ten years of a model of aesthetic appreciation and aesthetic judgments: the aesthetic episode—developments and challenges in empirical aesthetics. Br. J. Psychol. 105, 443–464. doi: 10.1111/bjop.12084, 25280118

[ref9003] Locher P. J. (2012). “Empirical investigation of an aesthetic experience with art,” In *Aesthetic science: Connecting minds, brains, and experience*. Eds. A. P. Shimamura and S. E. Palmer. (Oxford University Press), 163–188. doi: 10.1093/acprof:oso/9780199732142.003.0007

[ref11] Locher P. Krupinski E. A. Mello-Thoms C. Nodine C. F. (2007). Visual interest in pictorial art during an aesthetic experience. Spat. Vis. 21, 55–77. doi: 10.1163/156856808782713762, 18073051

[ref12] Locher P. Smith L. Smith J. (2001). The influence of presentation format and viewer training in the visual arts on the perception of pictorial and aesthetic qualities of paintings. Perception 30, 449–465. doi: 10.1068/p3008, 11383192

[ref9004] Massaro D. Savazzi F. Di Dio C. Freedberg D. Gallese V. Gilli G. . (2012). When art moves the eyes: A behavioral and eye-tracking study. Perception, 40, 747–760. doi: 10.1371/journal.pone.0037285 PMC3356266 22624007

[ref13] Mitrovic A. Hegelmaier L. M. Leder H. Pelowski M. (2020). Does beauty capture the eye, even if it's not (overtly) adaptive? A comparative eye-tracking study of spontaneous attention and visual preference with VAST abstract art. Acta Psychol. 209:103133. doi: 10.1016/j.actpsy.2020.103133, 32717655

[ref14] Nojo Y. (2026). Early depth engagement in art perception: visual dynamics and aesthetic experience. Front. Psychol. 10.3389/fpsyg.2026.1781822 PMC13130483 42077321

[ref15] Nojo Y. Chan A. B. (2025). Reading an artist’s intention from the composition (RAIC): eye movements and aesthetic experience in Japanese woodblock prints. Front. Psychol. 16:1644803. doi: 10.3389/fpsyg.2025.1644803, 41312285 PMC12648216

[ref16] Nojo Y. Maekawa T. Sato Y. Ueda K. (2023). Relating aesthetic-value judgment to perception: an eye-tracking and computational study of Japanese art Ukiyo-e. Proceedings of the Annual Meeting of the Cognitive Science Society, 45, 2282–2288.

[ref17] Pelowski M. Markey P. S. Förster M. Gerger G. Leder H. (2017). Move me, astonish me… delight my eyes and brain: the Vienna integrated model of top-down and bottom-up processes in art perception (VIMAP) and corresponding affective, evaluative, and neurophysiological correlates. Phys Life Rev 21, 80–125. doi: 10.1016/j.plrev.2017.02.003, 28347673

[ref18] Pelowski M. Markey P. S. Lauring J. O. Leder H. (2016). Visualizing the impact of art: an update and comparison of current psychological models of art experience. Front. Hum. Neurosci. 10:160. doi: 10.3389/fnhum.2016.00160, 27199697 PMC4844603

[ref19] Ratcliff R. McKoon G. (2008). The diffusion decision model: theory and data for two-choice decision tasks. Neural Comput. 20, 873–922. doi: 10.1162/neco.2008.12-06-420, 18085991 PMC2474742

[ref20] Shimojo S. Simion C. Shimojo E. Scheier C. (2003). Gaze bias both reflects and influences preference. Nat. Neurosci. 6, 1317–1322. doi: 10.1038/nn1150, 14608360

[ref21] Silvia P. J. (2005). Emotional responses to art: from collation and arousal to cognition and emotion. Rev. Gen. Psychol. 9, 342–357. doi: 10.1037/1089-2680.9.4.342

[ref22] Smith L. F. Smith J. K. Tinio P. P. L. (2017). Time spent viewing art and reading labels. Psychol. Aesthet. Creat. Arts 11, 77–85. doi: 10.1037/aca0000070

