# Supplementary material for: Phases of aesthetic judgment in art perception
Source: Front Psychol. 2026 Apr 22;17:1785560. doi: 10.3389/fpsyg.2026.1785560 (PMC13143710; doi:10.3389/fpsyg.2026.1785560)
Supplement: Supplementary file 1 [file Data_Sheet_1.PDF]

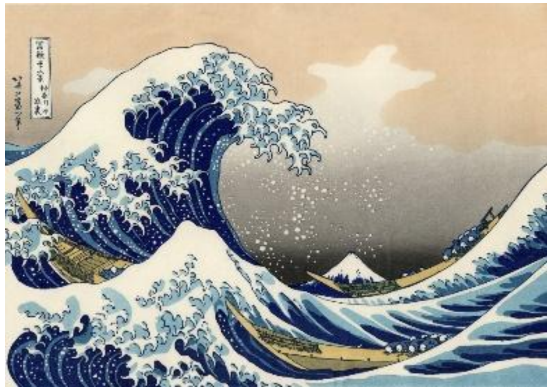
GreatWave

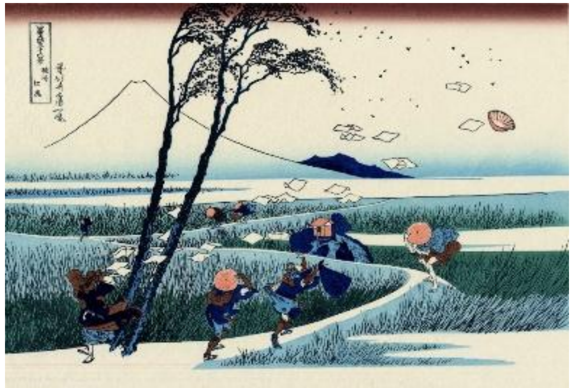
Ejiri

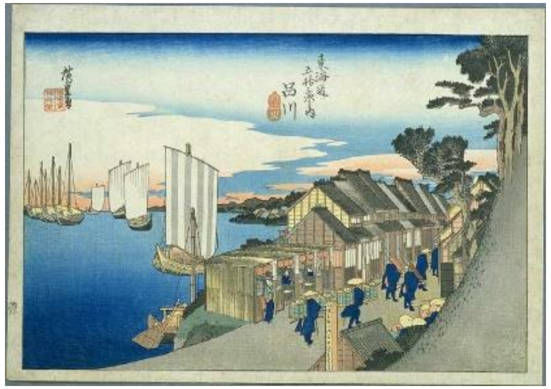
Shinagawa

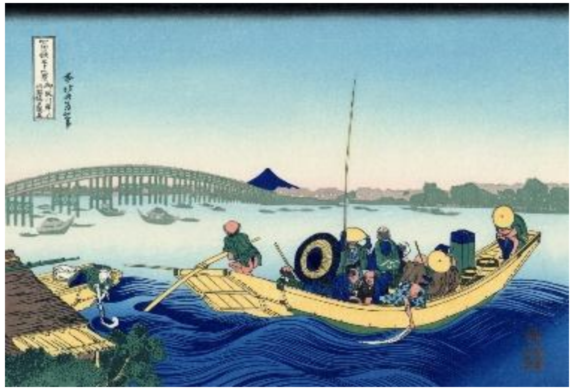
Ryogokubashi

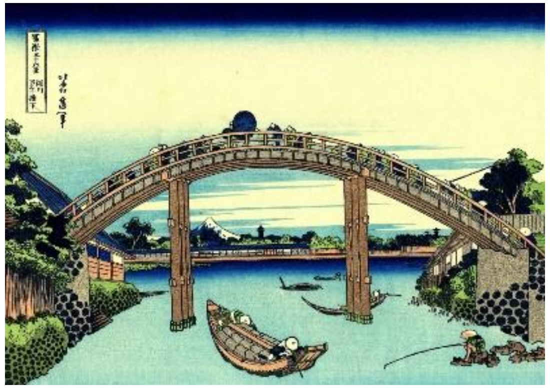
Mannenbashi

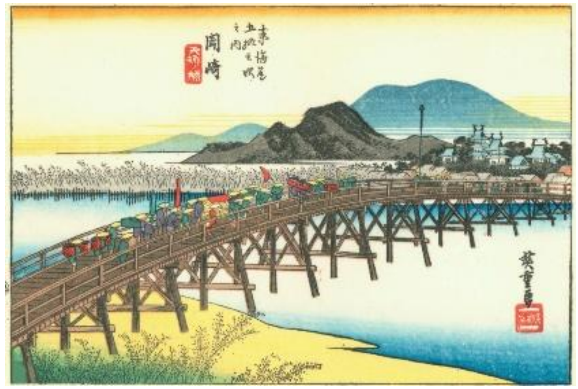
Okazaki

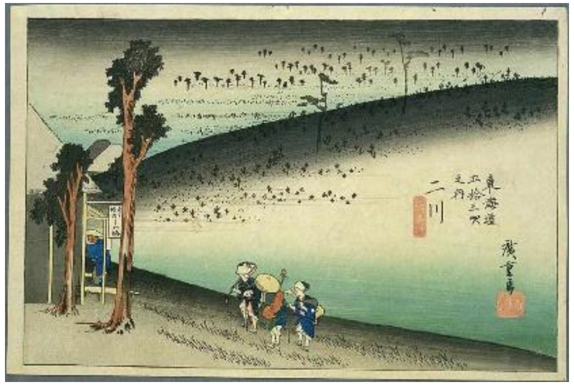
Futagawa

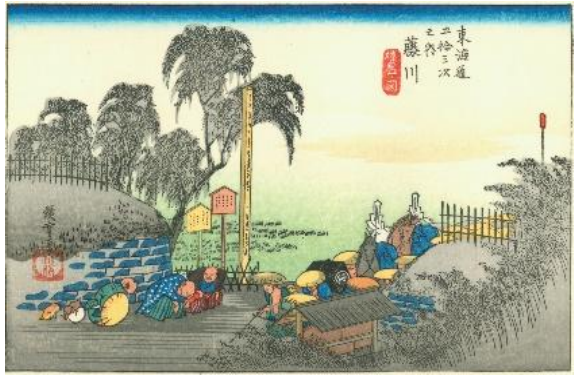
Fujikawa

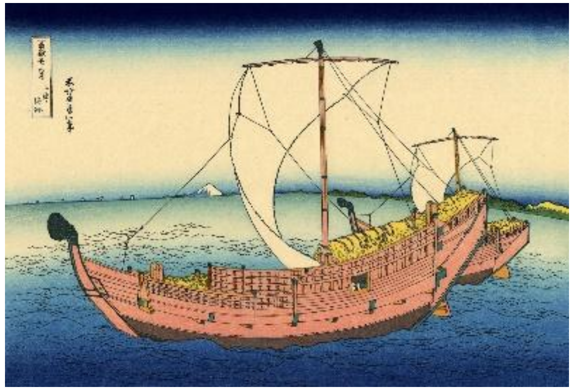
Kazusa

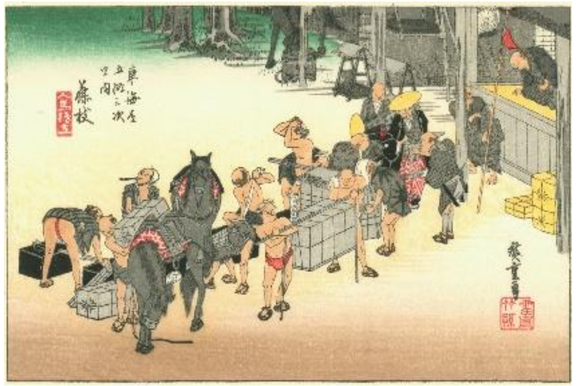
Fujieda

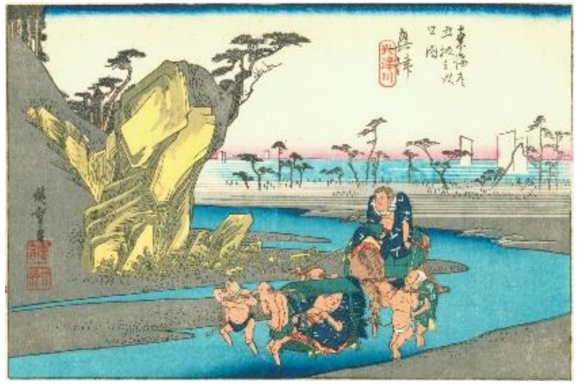
Okitsu

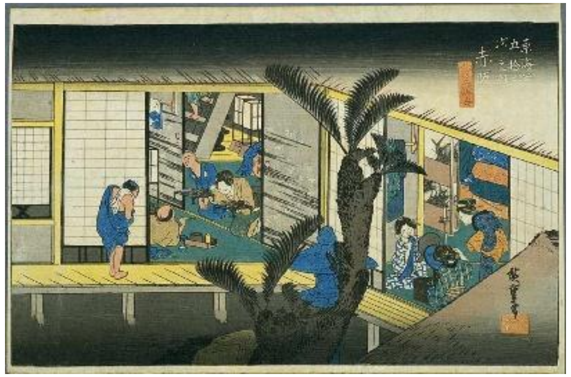
Akasaka
