# Supplementary figures and images for: Phases of aesthetic judgment in art perception
Source: Front Psychol. 2026 Apr 22;17:1785560. doi: 10.3389/fpsyg.2026.1785560 (PMC13143710; doi:10.3389/fpsyg.2026.1785560)

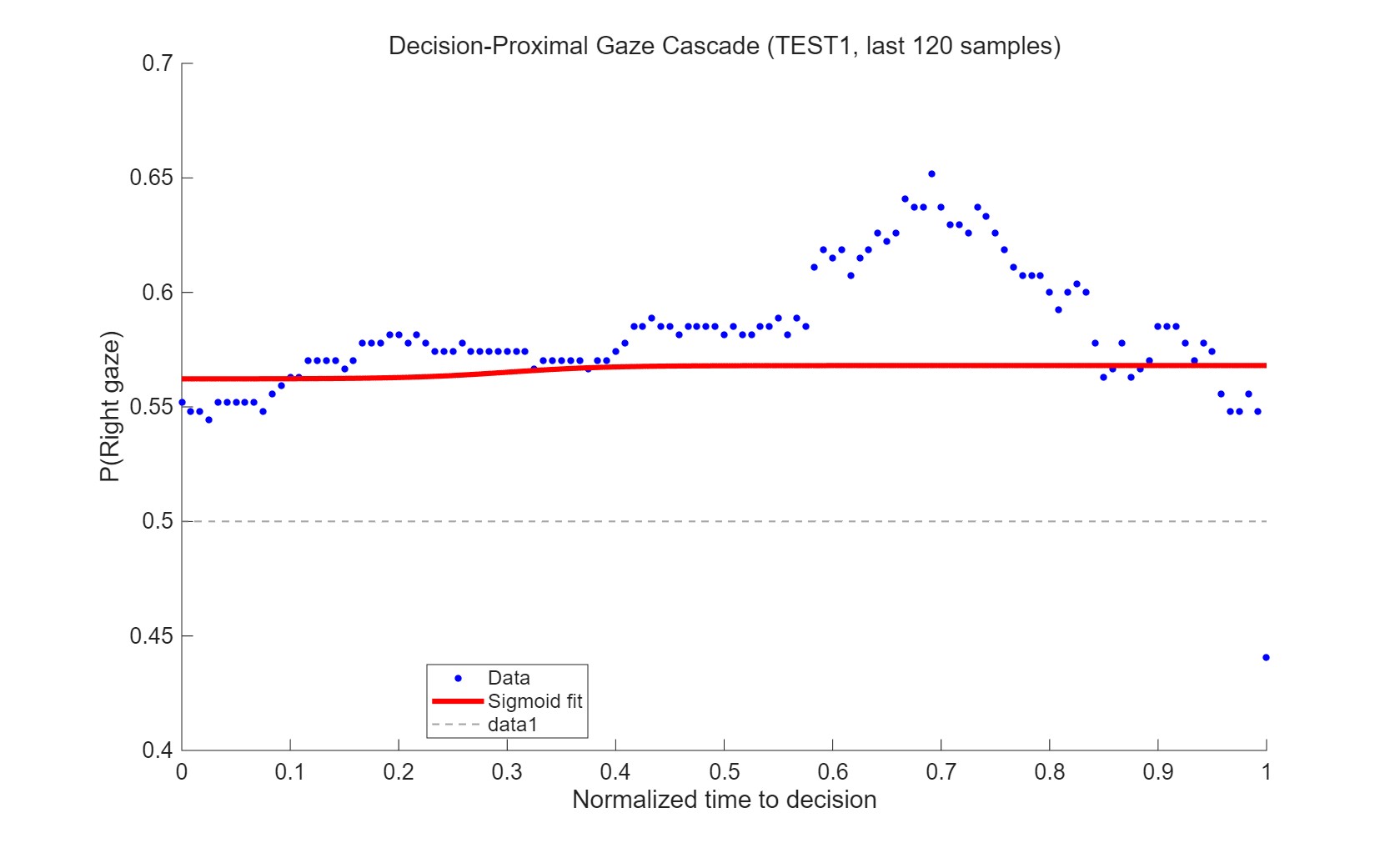

Supplement: Supplementary file 2 [file Image_1.jpeg]

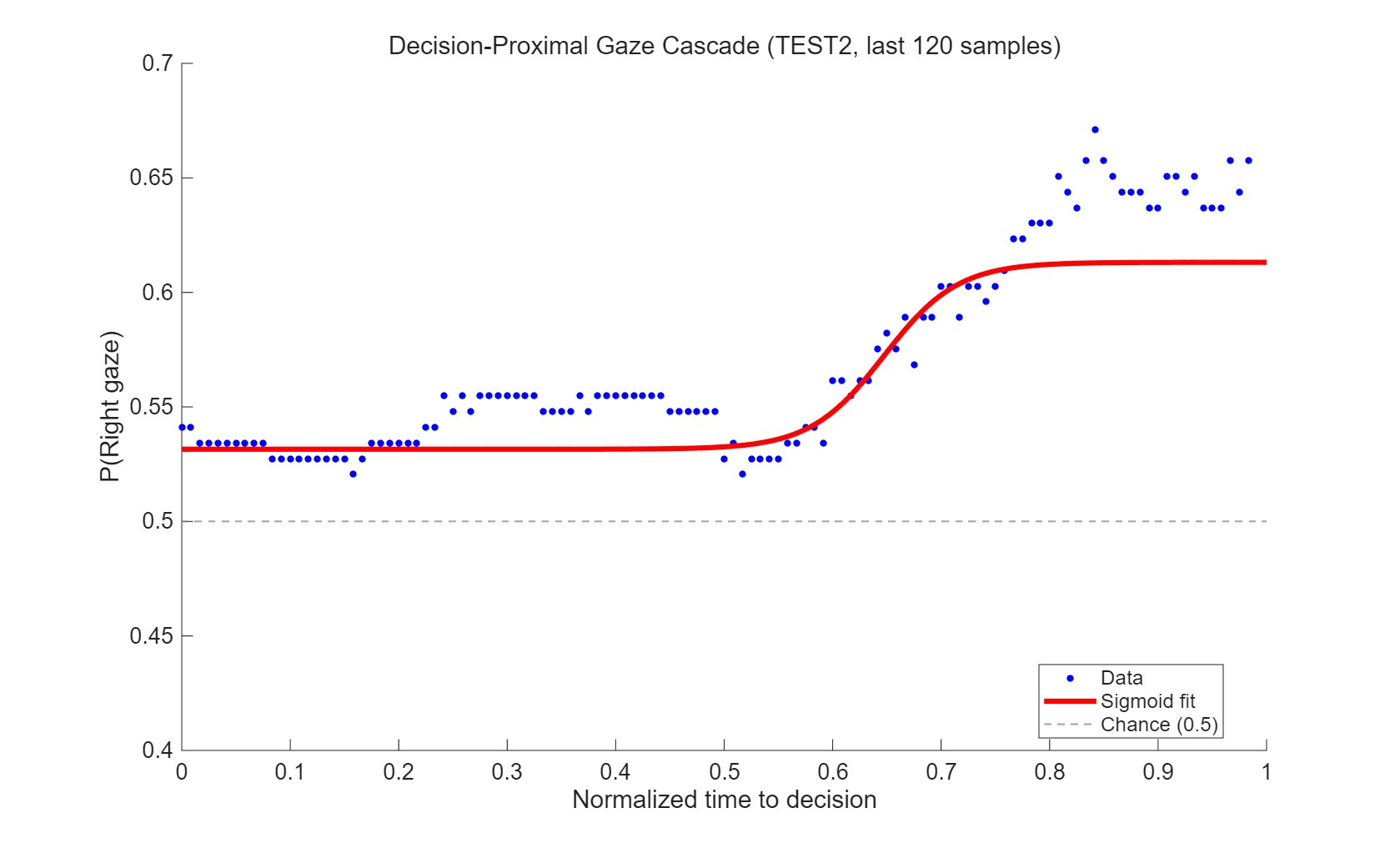

Supplement: Supplementary file 3 [file Image_2.jpeg]

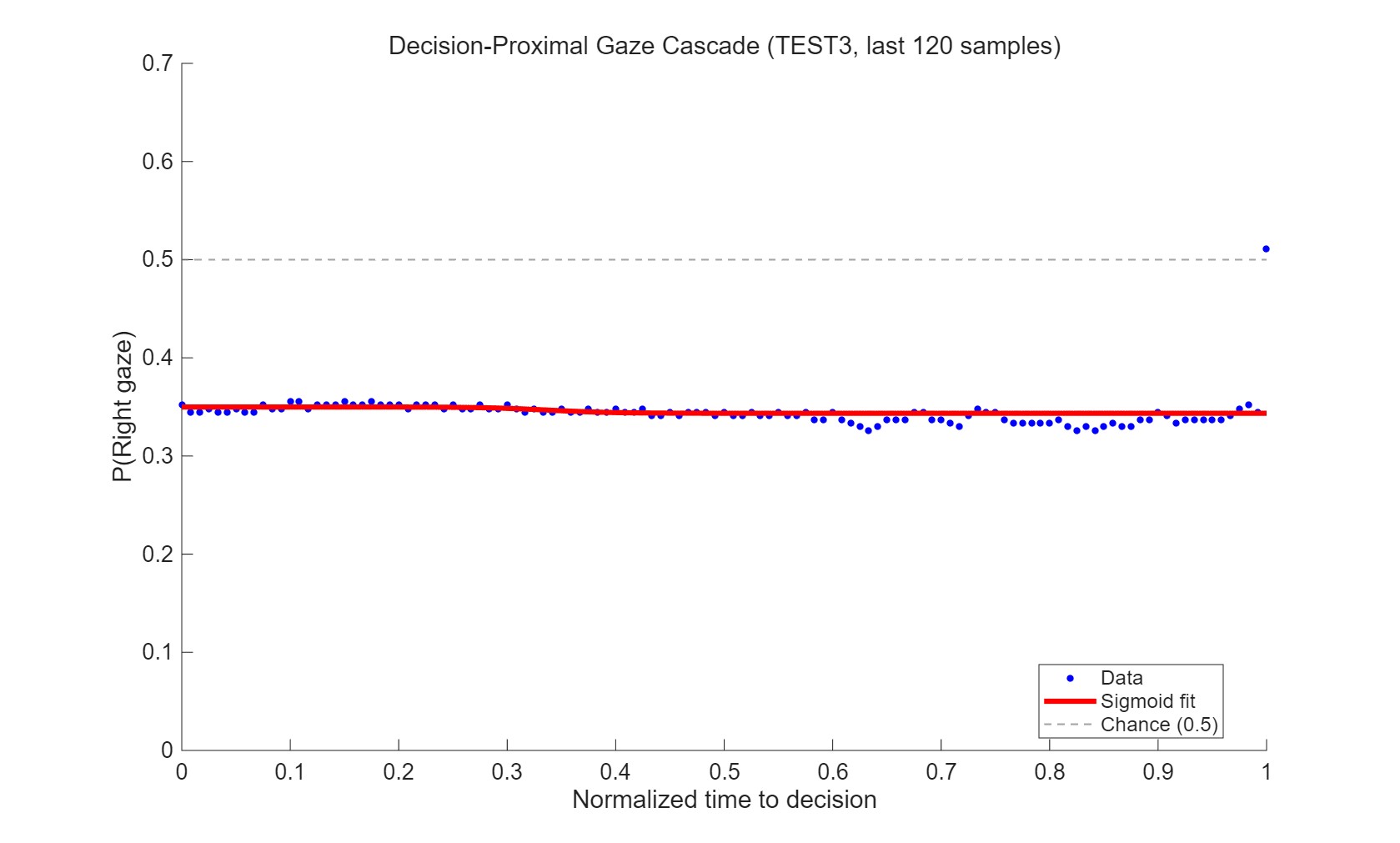

Supplement: Supplementary file 4 [file Image_3.jpeg]

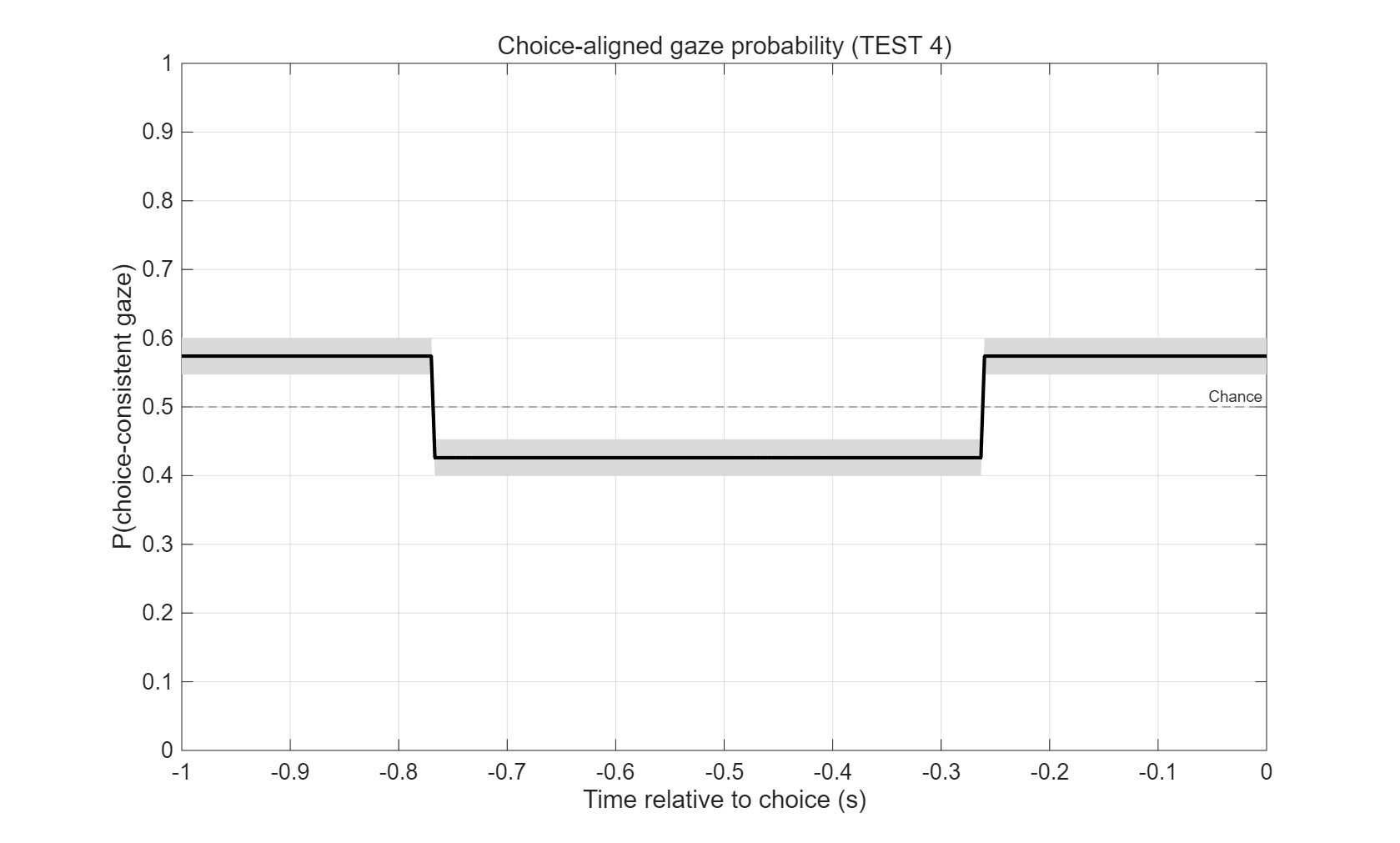

Supplement: Supplementary file 5 [file Image_4.jpeg]
